# Dentists Fees

**Published:** 1853-01

**Authors:** C. P. Culver

**Affiliations:** New Castle, Ky.


*Dentists Fees.* By Dr. C. P. Culver, New Castle, Ky.

There is no one profession within the state of Kentucky, wherein there exists such a wide difference in their fees, for like operations, as in that of dental surgery. This want of uniformity in their charges, originates from the want of efficient and skillful operators. There is, perhaps, no profession, from which the public have suffered such gross impositions, as that of dentistry.

Kentucky is infested with a set of *scullions*, who are prowling through the country, clamorous in their denunciations of what they are *pleased* to call, (*with all due defference*

*to the pecuniary interest of the dear people*,) "exhorbitant" fees, charged by the more skillful and local operators. The consequence is, that it is driving from the profession, men, who, from their knowledge and practical experience, are ornaments to their calling, and blessings to society.

Notwithstanding the impositions daily practiced by these *quacks* of the profession, and the direful consequences growing out of their mal-practice, the public at large have, from their associations with these men, partaken of the same spirit; and refuse to patronize a local and competent operator, upon the plea, that his bills are "*exhorbitant*;" choosing, rather to risk their chances of procuring an itinerant *cobler* who will extract for a *dime*, and fill *indiscriminately* for *fifty cents*.

Not only the good of the public, but justice and the interest of the profession, require that the dentist, skilled in a knowledge of his business, should require a liberal compensation for services rendered. This rule should hold good in all the associations of life, particularly the professions; as their success depends alone upon the skill and prudence with which they manage and dispose of business entrusted to their charge.

"No man can afford to spend the time and money necessary to obtain a thorough dental education, and avail himself of every facility to enable him to practice dentistry to the best advantage, unless he can receive for his operations what may seem to those who consider only the time spent in performing them, a high price.

No dentist, however high his reputation, has any right to demand exhorbitant prices; but every one who desires to see the interest of the profession advanced, should feel bound to take a firm stand for reasonable remuneration.

Does not the experience of every practitioner prove, that, unless he be well remunerated, he cannot do justice to those operated upon, without doing injustice to himself.

We have had enough *dental empiricism* practiced in our state by men who have no higher object in view, than the *almighty dollar;* caring nothing for the reputation of the profession; but alone, for the accumulation of the profits arising therefrom, however dishonestly obtained. The profession, as well as the *public*, should look to these impositions, and by every honorable effort, see that these *pirates* receive their just retribution; and the public enlightened as to the consequences arising from their mal-practice; and in this way, and no other, we shall rid the profession of a *curse*, and the public of a *nuisance*.

[Kentucky is not the only state where the condition of things described by Dr. Culver exists. There are ever those in the profession,—in all professions, who cry out against "exorbitant fees," "extravagant salaries," &c. There is, however, a special dishonesty among those claiming to be dentists, who pursue this course. Dishonest because such cannot use that amount of gold which is justly demanded in the operation;—dishonest because their patients are deceived and injured. It would be very easy demonstrating the fact that teeth cannot be filled with gold at 50 cts. per plug. We go farther, and say at 75 cts: We go farther, and say, in our cities, at $1. The idea of filling 25 to 30 cavities per day is ridiculous. No man regarding his professional reputation as worth any thing, would dare lay claim to such *skill*. There is, then, a maximum price below which good operations of this kind can never go. This maximum price will be regulated among the skilled in the profession in accordance with the necessary expenses incurred. We know that in towns where living is cheap, prices may justly be proportionably less. We never, therefore, look for uniformity of prices. We believe that the experience of all of our best operators is, that long experience does not enable them to plug teeth more rapidly. They cannot by dexterity thus acquired, increase the amount of their daily income at the same price. We see no reason why dentists should be confined to a mere subsistance. If they have talent and energy, why not allow it to be productive as in other professions. The fact is, the "public at large" will learn two lessons. First, that cheap dentistry is dear; and second, they will learn to appreciate good operations. Good operations will always ultimately bring reputation and patients; and this even among those who labor hard for a living. We presume that in no country are dental operations so common as in this; and we are glad to know that the poor as well as the rich, are often willing to remunerate well for the preservation of their teeth. We believe, that in every intelligent community, (in this country,) good operations will ultimately command fair and remunerating prices; and we doubt not, that ere long this will be the conviction of our friend in Kentucky.

We know of no way to remedy the evil now complained of, except by supplying the community with intelligent and skillful operators.—ED.]

